# Impulse Oscillometry Reveals Predominantly Mechanical Drivers of Small Airway Dysfunction in Obese Children

**DOI:** 10.3390/jcm15124480

**Published:** 2026-06-10

**Authors:** Ercan Yılmaz, Zeynep Yamancan Yılmaz, İsmail Dündar, Emine Çamtosun, Erdem Topal

**Affiliations:** 1 Department of Pediatric Allergy and Immunology, Faculty of Medicine, Inonu University, 44000 Malatya, Turkey; ercanyilmz44@gmail.com; 2 Department of Pediatric Endocrinology, Faculty of Medicine, Inonu University, 44000 Malatya, Turkey; zynpymncan@hotmail.com (Z.Y.Y.); ismail.dundar@inonu.edu.tr (İ.D.); epurcuklu@gmail.com (E.Ç.)

**Keywords:** insulin resistance, impulse oscillometry, small airway disease, obesity

## Abstract

**Background:** Childhood obesity can cause changes in respiratory mechanics, particularly in the peripheral airways. This study aimed to evaluate impulse oscillometry (IOS) parameters in obese children and to investigate the relationship between small airway function and metabolic indicators. **Methods:** A total of 135 children and adolescents (56.3% female) were included in the study. Seventy participants were obese, and 65 were in the normal-weight control group. IOS parameters (R5, R20, R5–R20, X5, X20, AX, Fres) were compared between the two groups. Obese participants were further divided into subgroups based on HOMA-IR levels, with and without metabolic disorders. The relationships between IOS parameters and body mass index z-score, HOMA-IR, and HbA1c were evaluated using correlation analysis. **Results:** The R5 value was significantly higher in the obese group compared to the control group (*p* = 0.01). The R20 parameter and R5–R20 parameter were markedly increased in the obese group (*p* = 0.03 and *p* < 0.001, respectively). The AX value was also significantly higher in the obese group (*p* = 0.02). No significant differences were found between the groups in terms of X5, X20, and Fres (*p* > 0.05). In the metabolic sub-analysis of the obese group, IOS parameters did not differ according to metabolic status. However, a weak positive correlation was found between HOMA-IR and R5–R20 (r = 0.257, *p* = 0.031) and AX (r = 0.290, *p* = 0.015). A moderate positive relationship was found between BMI z-score and R5–R20 (r = 0.424, *p* < 0.001) and AX (r = 0.383, *p* = 0.01). **Conclusions:** Small airway dysfunction was detected in obese children. The findings suggest that mechanical factors may be more dominant than metabolic factors in peripheral airway involvement.

## 1. Introduction

Childhood obesity has reached epidemic proportions worldwide and now constitutes a significant public health problem due to its consequences, affecting many organ systems. Beyond its cardiometabolic complications, obesity is increasingly being linked to changes in respiratory mechanics and impaired lung function, even at an early age [1,2]. Recent pediatric studies have confirmed that, although spirometric values are generally normal in obese children, there is an increase in total and peripheral airway resistance as measured by impulse oscillometry (IOS) [3,4,5].

The effect of obesity on lung function has traditionally been attributed to mechanical factors; excessive thoracoabdominal adiposity reduces the elasticity of the chest wall, decreases functional residual capacity, leads to premature airway closure and increases small airway resistance [2,6]. However, current evidence suggests that obesity represents a complex systemic inflammatory condition rather than a purely mechanical disorder. Adipose tissue acts as an endocrine organ secreting pro-inflammatory mediators such as interleukin-6, tumor necrosis factor-alpha and leptin, and these substances may contribute to airway inflammation and remodeling [7].

Recent studies have reinforced the view that obesity-related airway dysfunction cannot be explained by mechanical loading alone. Pediatric IOS-based studies have demonstrated that obese children have significantly higher R5–R20 and AX values compared to their normal-weight peers; this indicates that the small airways are affected at an early stage, even in the absence of a pronounced clinical respiratory disease [3,4,8].

In parallel, metabolic dysregulation -particularly insulin resistance- has emerged as a significant modifier of respiratory physiology. Insulin resistance has been associated with airway smooth muscle proliferation, altered bronchial reactivity, and enhanced inflammatory signaling pathways [5,9,10]. Importantly, recent clinical evidence suggests that metabolic abnormalities may contribute to airway dysfunction independently of the effects of adiposity [9].

The distinction between metabolically healthy obesity (MHO) and metabolically unhealthy obesity (MUO) has therefore gained increasing attention. MUO is characterized by insulin resistance and systemic inflammation and has been associated with worse cardiometabolic outcomes. However, its impact on small airway functions remains poorly understood, particularly in pediatric populations where available data are limited and heterogeneous [8,11].

Impulse oscillometry (IOS) offers a sensitive and non-invasive method for detecting early-stage small airway dysfunction during normal breathing. Studies have demonstrated that parameters such as R5–R20 and AX detect peripheral airway abnormalities that cannot be identified by spirometry [4,5,12]. Recent pediatric studies have further highlighted that IOS parameters are influenced by both obesity and metabolic status; however, the findings are inconsistent, and the number of studies is limited [3,8]. Therefore, further studies are required to clarify the independent contributions of mechanical and metabolic factors to small airway dysfunction in obese children, particularly by classifying patients according to metabolic phenotype (MHO and MUO).

This study aims to evaluate small airway function in obese children and adolescents using IOS and to investigate the contribution of metabolic dysfunction accompanying obesity to peripheral airway dysfunction. In this context, the objective is to determine whether mechanical loading or the metabolic/inflammatory process plays a more dominant role in small airway changes.

## 2. Materials and Methods

### 2.1. Study Design and Patient Population

This study was designed as a prospective, cross-sectional, case–control study. Children and adolescents aged 8–17 years who visited the pediatric endocrinology outpatient clinic were included in the study. The study group consisted of obese individuals, while the control group consisted of healthy normal-weight children.

Obesity was defined according to World Health Organization (WHO) criteria as a body mass index (BMI) ≥ 95th percentile for age and sex [1]. Normal weight was defined as a BMI between the 5th and 85th percentiles. Height was measured using a stadiometer, and body weight was measured using a calibrated digital scale. BMI was calculated by dividing weight in kilograms by height in meters squared (kg/m^2^). BMI Z-scores were calculated according to age- and sex-specific reference standards of the World Health Organization, and participants with a BMI Z-score > +2 were classified as obese.

Children with a history of chronic lung disease, atopic disease (including asthma, allergic rhinitis), congenital heart disease, neuromuscular disorders, prematurity, or respiratory tract infection within the preceding four weeks were excluded.

### 2.2. Metabolic Assessment and Definitions

Venous blood samples were obtained from all participants after an overnight fast of at least 8–12 h. Blood samples were collected between 08:00 and 10:00 a.m. to minimize the effects of circadian variation. Serum fasting plasma glucose (FPG) levels were measured using the hexokinase method, and fasting insulin levels were determined by a chemiluminescent immunoassay method. Insulin resistance was calculated using the Homeostasis Model Assessment of Insulin Resistance (HOMA-IR) method [13,14,15]. All analyses were performed in the same certified laboratory following standardized procedures. HOMA-IR was calculated using the following formula:

HOMA-IR = [fasting insulin (µIU/mL) × fasting glucose (mg/dL)]/405

For glucose values expressed in mmol/L, the formula used was:

HOMA-IR = [fasting insulin (µIU/mL) × fasting glucose (mmol/L)]/22.5

Pubertal status was assessed according to Tanner staging by a trained clinician. Based on established pediatric cut-off values, HOMA-IR > 2.5 in prepubertal children and >3.16 in pubertal children were considered indicative of insulin resistance.

Participants in the obese group were further categorized into two subgroups: metabolically normal obese (MNO) and metabolically unhealthy obese (MUO). Metabolic disorder (MUO) was defined as the presence of insulin resistance based on the HOMA-IR thresholds described above.

Glycated hemoglobin (HbA1c) levels were measured using high-performance liquid chromatography (HPLC), and values were expressed as percentages (%). HbA1c was included as an additional marker of long-term glycemic control.

### 2.3. Assessment of Airway Functions Using Impulse Oscillometry

Airway resistance and reactance were assessed using an impulse oscillometry system (IOS) (Jaeger MasterScreen IOS, CareFusion, Yorba Linda, CA, USA). The device was calibrated daily prior to testing using a standard 3 L syringe and reference resistance, in accordance with the manufacturer’s instructions. All measurements were performed in accordance with the technical standards for respiratory oscillometry published by the European Respiratory Society (ERS) and supported by the American Thoracic Society [12]. Participants were instructed to avoid heavy meals, vigorous exercise, and short-acting bronchodilators for at least 4–6 h prior to testing. Measurements were conducted in a quiet environment with participants in a seated position, wearing a nose clip and maintaining a neutral head position. To minimize upper airway artifact, participants firmly supported their cheeks and the floor of the mouth with their hands.

During the procedure, subjects were asked to breathe normally (tidal breathing) through a mouthpiece connected to the IOS device for approximately 30–45 s, ensuring a tight seal around the mouthpiece. At least three technically acceptable measurements were obtained from each participant. Measurements were considered acceptable if they met the following criteria: absence of artifacts such as coughing, swallowing, vocalization, air leakage, or irregular breathing; a stable tidal breathing pattern; and coherence values of ≥0.80 at 5 Hz and ≥0.90 at frequencies ≥ 10 Hz. The mean values of the acceptable measurements were used for analysis.

The IOS parameters evaluated included total airway resistance at 5 Hz (R5), central airway resistance at 20 Hz (R20), and the difference between R5 and R20 (R5–R20), which reflects peripheral (small airway) resistance. Reactance parameters included reactance at 5 Hz (X5), reactance area (AX), and resonance frequency (Fres). Negative X5 values indicate reduced elastic recoil and peripheral airway involvement.

All IOS parameters were expressed as absolute values and, where available, as percentages of predicted values based on age-, sex-, height-, and ethnicity-specific reference equations. Small airway dysfunction was defined by elevated R5–R20, increased AX, and/or increased Fres values beyond the upper limit of normal (ULN), according to reference standards.

### 2.4. Statistical Analysis

Statistical analysis was performed using SPSS Statistics (v.21.0; IBM Corp., Armonk, NY, USA). Normality was assessed using the Kolmogorov–Smirnov test. Descriptive statistics were expressed as frequency and percentage for categorical variables, whereas the mean ± SD was used for quantitative data. Categorical variables were compared using the Pearson’s chi-square test or Fisher’s exact test, and quantitative variables were compared using the *t*-test and Mann–Whitney U test. Relationships between metabolic parameters (HOMA-IR, HbA1c, BMI Z-score) and IOS parameters were evaluated using Spearman correlation analysis. Two-tailed *p*-values < 0.05 were considered statistically significant.

Local ethics committee approval was obtained for the study, and written informed consent was obtained from parents, and assent was obtained from participants.

## 3. Results

### 3.1. Demographic Characteristics of Patients

A total of 135 children and adolescents were included in the study. Seventy-six (56.3%) of the participants were female, and the mean age was 13.2 ± 2.9 years. Seventy were obese, while 65 were in the normal-weight control group. No statistically significant difference was found between the obese and control groups in terms of age and sex distribution (*p* > 0.05). Similarly, no significant difference was found between the groups in terms of smoking exposure rates (*p* = 0.298) (Table 1).

### 3.2. Comparison of Impulse Oscillometry Parameters Between Obese and Control Groups

When R5 values were compared between the obese group and the healthy control group, the R5 value in the obese group (0.517 ± 0.185 kPa·s/L) was found to be higher than that in the control group (0.420 ± 0.181 kPa·s/L), and this difference was statistically significant (*p* = 0.01). R20 values were higher in the obese group (0.312 ± 0.095 kPa·s/L) compared to the control group (0.284 ± 0.106 kPa·s/L), and the difference was statistically significant (*p* = 0.03). The R5–R20 difference, reflecting small airway resistance, was found to be significantly higher in the obese group (0.215 ± 0.127 kPa·s/L) compared to the control group (0.136 ± 0.096 kPa·s/L), and this difference was highly statistically significant (*p* < 0.001). Although X5 values were more negative in the obese group (−0.121 ± 0.111 kPa·s/L) compared to the control group (−0.088 ± 0.075 kPa·s/L), this difference was not statistically significant (*p* = 0.159). X20 values were similar in the obese group (−0.016 ± 0.058 kPa·s/L) and the control group (−0.013 ± 0.067 kPa·s/L), and the difference between them was not statistically significant (*p* = 0.484). The AX value was found to be significantly higher in the obese group (1.660 ± 1.149 kPa/L) compared to the control group (1.150 ± 0.958 kPa/L) (*p* = 0.02). When Fres values were compared, no significant difference was found between the obese group (19.949 ± 5.522 Hz) and the control group (20.203 ± 6.640 Hz) (*p* = 0.890) (Table 1).

Overall, significant increases were observed in the obese group, particularly in resistance parameters reflecting small airway function (R5, R20, and R5–R20) and in the AX value; no significant difference was found between the two groups in terms of reactance parameters and resonance frequency.

### 3.3. Comparison and Correlation Analysis Based on Metabolic Status in Obese Patients

Obese participants were divided into two subgroups based on HOMA-IR levels: those with metabolic abnormalities and those without. When the groups were compared in terms of small airway function parameters, no statistically significant difference was found in R5–R20 values (*p* = 0.157). Similarly, no significant difference was observed between the groups in terms of the AX parameter, which reflects peripheral airway reactance (*p* = 0.394). Evaluation of other impulse oscillometry (IOS) parameters also revealed no statistically significant differences based on metabolic status (*p* > 0.05 for all comparisons) (Table 2).

In the correlation analysis performed, a weak positive and statistically significant correlation was found between HOMA-IR, which is considered an indicator of metabolic dysfunction in obese patients, and the R5–R20 parameter, which is an indicator of small airway dysfunction (Spearman r = 0.257, *p* = 0.031). Similarly, a weak positive and statistically significant relationship was found between HOMA-IR and the AX parameter, which reflects peripheral airway reactance (Spearman r = 0.290, *p* = 0.015). When evaluating the relationship between HbA1c levels and small airway parameters, no significant correlation was found between HbA1c and R5–R20 (Spearman r = 0.066, *p* = 0.584). A weak positive correlation was observed between HbA1c and AX, but the clinical relevance of this association was limited (Spearman r = 0.238, *p* = 0.047).

On the other hand, a moderate positive and statistically significant correlation was found between R5–R20, an indicator of small airway dysfunction, and BMI z-score (Spearman r = 0.424, *p* < 0.001). Similarly, a significant relationship was found between the AX parameter and BMI z-score (Spearman r = 0.383, *p* = 0.01).

## 4. Discussion

This study demonstrates that small airway function is significantly impaired in obese children and adolescents. The significantly higher R5, R5–R20, and AX parameters in the obese group compared to the control group, as assessed by impulse oscillometry, reveal that obesity has a marked effect on peripheral airways. Our findings further suggest that the impairment in airway function is predominantly driven by mechanical rather than metabolic factors.

The relationship between obesity and small airway dysfunction has been increasingly recognized in the recent literature, with accumulating evidence highlighting both mechanical and inflammatory mechanisms. The effects of obesity on lung mechanics are primarily explained by decreased thoracic compliance, cranial displacement of the diaphragm due to excess abdominal adiposity, reduced functional residual capacity (FRC), and premature closure of peripheral airways during tidal breathing [16,17]. These mechanical constraints result in ventilation inhomogeneity and a disproportionate impact on the small airways, which are highly sensitive to changes in lung volume.

In addition, increased adipose tissue in the thoracic and abdominal compartments exerts compressive forces on the lungs and chest wall, limiting lung expansion and promoting airway narrowing, particularly at low lung volumes. This effect is further exacerbated in the supine position and contributes to early airway closure and gas trapping [18,19,20]. Beyond mechanical factors, obesity is also characterized by a chronic low-grade systemic inflammatory state, with elevated levels of adipokines and pro-inflammatory cytokines such as leptin, TNF-α, and IL-6. These mediators may contribute to airway inflammation, remodeling, and increased airway hyperresponsiveness, thereby worsening small airway dysfunction [19,20].

These pathophysiological processes are reflected in impulse oscillometry parameters. Increased R5–R20 values indicate elevated peripheral airway resistance, while higher AX values reflect increased reactance and reduced compliance of the distal lung regions. The findings of our study, showing elevated R5–R20 and AX values in obese individuals, are consistent with these mechanisms and align with recent reports emphasizing the sensitivity of oscillometry in detecting early small airway impairment [12,21]. Collectively, these findings support the concept that obesity adversely affects small airway function through a combination of mechanical restriction and systemic inflammation, potentially increasing the risk of respiratory morbidity.

The primary objective of our study was to investigate whether small airway dysfunction is solely attributable to the mechanical burden of obesity or whether the metabolic disorder (insulin resistance) accompanying obesity also plays a role in this process. When the obese group was divided into subgroups based on HOMA-IR levels, no significant difference was found in IOS parameters between patients with and without metabolic disorders. This finding suggests that metabolic status does not have a significant discriminatory effect on small airway function.

Although obesity is characterized by systemic low-grade inflammation and it has been suggested that this inflammation may contribute to airway inflammation [22,23], our study found only a weak correlation between HOMA-IR and small airway parameters. Similarly, no strong and consistent relationship was demonstrated between HbA1c and IOS parameters. This suggests that the effect of metabolic dysfunction on peripheral airways may be limited.

Moderate correlations were identified between body mass index (BMI) z-score and both R5–R20 and AX, supporting the notion that increasing degrees of adiposity are closely associated with worsening small airway function. These findings suggest that as BMI increases, peripheral airway resistance and reactance abnormalities become more pronounced, reflecting early impairment in distal airway mechanics [6]. Notably, the relationship between BMI z-score and small airway parameters was stronger than that observed with HOMA-IR, indicating that anthropometric measures of obesity may have a more direct impact on lung function than metabolic dysregulation alone. This distinction is important, as it implies that the mechanical burden of excess body weight may play a more dominant role than insulin resistance in shaping respiratory physiology.

From a pathophysiological perspective, increasing BMI is associated with progressive reductions in functional residual capacity (FRC) and expiratory reserve volume (ERV), both of which are critical determinants of small airway patency. At lower lung volumes, the tethering forces that normally keep small airways open are diminished, predisposing them to early closure and increasing ventilation heterogeneity [6,16]. This mechanical disadvantage becomes more evident with higher BMI z-scores, explaining the stronger correlations observed with oscillometric indices such as R5–R20 and AX. In contrast, while HOMA-IR reflects systemic metabolic status and insulin resistance, its indirect relationship with lung mechanics may account for the weaker associations observed in our analysis.

The literature similarly reports that the relationship between the degree of obesity and alterations in lung volumes and airway resistance is more pronounced than that with metabolic markers such as insulin resistance or lipid profiles [16,19]. Furthermore, studies in both pediatric and adult populations have demonstrated that measures of central adiposity, including waist circumference and BMI z-score, correlate more strongly with reductions in lung volumes and increases in airway resistance than biochemical indicators of metabolic dysfunction [18,24]. These findings reinforce the concept that excess adipose tissue exerts direct mechanical constraints on the respiratory system, particularly affecting the small airways.

When these findings are considered together, it becomes evident that small airway dysfunction in obese individuals is likely driven predominantly by the mechanical effects of obesity, rather than metabolic disturbances alone. The stronger association between BMI z-score and oscillometric parameters such as R5–R20 and AX underscores the sensitivity of small airways to changes in lung volume and chest wall mechanics. While metabolic factors may still contribute through systemic inflammation and airway remodeling, the primary driver appears to be the physical impact of excess body mass on respiratory mechanics. This highlights the importance of weight management strategies in preserving small airway function and preventing long-term respiratory morbidity.

If metabolic or inflammatory mechanisms played a dominant role in the development of small airway dysfunction, it would be expected that more pronounced differences in impulse oscillometry (IOS) parameters and stronger correlations between groups stratified by insulin resistance status. In particular, obese individuals with elevated HOMA-IR values would be anticipated to exhibit significantly higher R5–R20 and AX measurements compared to those without insulin resistance, reflecting a greater degree of airway inflammation and remodeling. However, no such distinction was observed in our study, as IOS parameters were comparable between HOMA-IR–positive and –negative obese individuals. This finding suggests that insulin resistance alone may not be a key determinant of small airway dysfunction in this population.

From a mechanistic standpoint, metabolic dysfunction-characterized by insulin resistance, dyslipidemia, and systemic inflammation- is known to contribute to airway inflammation through circulating cytokines such as interleukin-6 and tumor necrosis factor-alpha. These mediators may promote airway wall thickening, increased smooth muscle tone, and airway hyperresponsiveness [19,24]. Therefore, if these pathways were dominant, clearer physiological distinctions in IOS measurements would be expected between metabolically distinct obese subgroups. The absence of such differences in our findings does not support a primarily metabolic origin for a primarily metabolic or inflammatory origin of small airway impairment, at least in the early or moderate stages of obesity.

Taken together, the absence of significant IOS differences between HOMA-IR–defined subgroups in our study suggests that mechanical loading plays a more prominent role than inflammatory or metabolic effects in the pathophysiology of obesity-related small airway dysfunction. While metabolic and inflammatory pathways may still contribute, particularly in more advanced disease or in specific phenotypes, their impact appears secondary to the direct mechanical consequences of excess adiposity. These findings emphasize the importance of considering biomechanical factors when evaluating respiratory dysfunction in obesity and may have implications for targeted therapeutic strategies.

This study has certain limitations. Metabolic status was assessed using HOMA-IR and HbA1c, and biomarkers directly reflecting systemic inflammation associated with obesity were not examined. Furthermore, since lung volumes were not measured, the mechanical effect of obesity could not be directly quantified. The lack of systematic assessment and adjustment for key lifestyle and developmental factors such as physical activity, dietary habits, and pubertal status may have introduced residual confounding in the observed associations between metabolic and respiratory outcomes. Due to the limited sample size within the subgroups, an adequate events-per-variable ratio was not achieved for multivariable regression modeling; therefore, multivariable analyses were not performed to avoid potential overfitting. This may limit the ability to identify independent predictors of IOS parameters. Additionally, the single-center design and relatively small sample size limit the generalizability of the findings. However, our study makes an important contribution to the literature by comparatively addressing the relative contributions of mechanical and metabolic factors in obesity-related small airway dysfunction.

## 5. Conclusions

This study demonstrates that small airway dysfunction is present in obese children, as evidenced by increased R5–R20 and AX values measured by impulse oscillometry. While metabolic dysregulation, particularly insulin resistance, showed weak but significant correlations with small airway indices, metabolic phenotype–based subgrouping did not reveal significant differences, suggesting that mechanical factors related to obesity may play a more dominant role in peripheral airway involvement. The novelty of this study lies in the combined evaluation of IOS parameters with metabolic status in a pediatric population, contributing to the limited and heterogeneous literature in this field. However, the cross-sectional design, relatively small sample size, and longitudinal follow-up limit causal inference. Future prospective studies with larger cohorts are needed to clarify the temporal relationship between metabolic dysfunction and airway impairment and to determine whether early metabolic control may influence the progression of small airway dysfunction in obese children.

## Figures and Tables

**Table 1:** Demographic Characteristics and Distribution of Oscillometric Measurements in the Obese and Healthy Control Groups.

Variables	Obese Group (n: 70)	Healthy Control Group (n: 65)	*p* Value
Gender, female, *n* (%)	43 (61.4)	33 (50.8)	0.212
Age, mean ± SD, years	12.88 ± 2.92	13.58 ± 3.05	0.093
Exposure to cigarettes, *n* (%)	48 (68.6)	38 (58.5)	0.298
R5 (kPa·s/L), mean ± SD	0.517 ± 0.185	0.420 ± 0.181	0.01
R20 (kPa·s/L), mean ± SD	0.312 ± 0.095	0.284 ± 0.106	0.03
R5–R20 (kPa·s/L), mean ± SD	0.215 ± 0.127	0.136 ± 0.096	<0.001
X5 (kPa·s/L), mean ± SD	−0.121 ± 0.111	−0.088 ± 0.075	0.159
X20 (kPa·s/L), mean ± SD	−0.016 ± 0.058	−0.013 ± 0.067	0.484
AX (kPa/L), mean ± SD	1.660 ± 1.149	1.150 ± 0.958	0.02
Fres (Hz), mean ± SD	19.949 ± 5.522	20.203 ± 6.640	0.89

**Table 2:** Comparison of Demographic Characteristics and Impulse Oscillometry (IOS) Parameters in HOMA-IR Positive and Negative Groups.

IOS Parameters	HOMA-IR–Positive Group (n: 42)	HOMA-IR–Negative Group (n: 28)	*p* Value
Gender, female, *n* (%)	29 (69)	14 (50)	0.176
Age, mean ± SD, years	13.35 ± 2.77	12.17 ± 3.04	0.118
Exposure to cigarettes, *n* (%)	29(69)	19 (67.9)	1.0
BMI z-score, mean ± SD	2.723 ± 0.702	2.335 ± 0.447	0.016
R5 (kPa·s/L), mean ± SD	0.556 ± 0.226	0.491 ± 0.150	0.16
R20 (kPa·s/L), mean ± SD	0.313 ± 0.104	0.311 ± 0.09	0.688
R5–R20 (kPa·s/L), mean ± SD	0.245 ± 0.142	0.195 ± 0.112	0.157
X5 (kPa·s/L), mean ± SD	−0.115 ± 0.124	−0.131 ± 0.09	0.325
X20 (kPa·s/L), mean ± SD	−0.006 ± 0.056	−0.032 ± 0.059	0.51
AX (kPa/L), mean ± SD	1.962 ± 1.313	1.483 ± 0.993	0.394
Fres (Hz), mean ± SD	19.362 ± 5.237	20.830 ± 5.910	0.281

## Data Availability

The data presented in this study are available on request from the corresponding author.
